# Patient Experiences With Virtual Care During the COVID-19 Pandemic: Phenomenological Focus Group Study

**DOI:** 10.2196/42966

**Published:** 2023-05-01

**Authors:** Vernon R Curran, Ann Hollett, Emily Peddle

**Affiliations:** 1 Faculty of Medicine Memorial University St John's, NL Canada

**Keywords:** virtual care, focus group, patients, patient education, qualitative study, patient experience, health care system, digital literacy, rural community, technology adoption, COVID-19

## Abstract

**Background:**

Virtual care has expanded during the COVID-19 pandemic and enabled greater access and continuity of care for many patients. From a patient-oriented research perspective, understanding the patient experience with virtual care appointments is an important first step in identifying ways to better support patient use and satisfaction.

**Objective:**

The purpose of this qualitative study was (1) to explore patients’ experiences and perspectives with the adoption and use of virtual care during COVID-19 in Newfoundland and Labrador, Canada, and (2) identify the education and informational needs of patients to inform future strategies for supporting patient use of virtual care.

**Methods:**

Using a phenomenological approach, we conducted a focus group interview with a purposive sample of patient representatives representing a cross-section of the population of the province of Newfoundland and Labrador. Five patient representatives were recruited from the Newfoundland and Labrador Support Patient Advisory Council and participated in the focus group. The focus group was conducted in February 2022 via videoconferencing technology. Using thematic analysis, we identified several recurrent themes that described respondents’ experiences with the use of virtual care during COVID-19, as well as their perceptions of education and informational needs to support more effective patient use of virtual care.

**Results:**

Respondents felt that virtual care is a beneficial addition to the health care system, enabling greater convenience and access to health care services. Key barriers and challenges in adopting and using virtual care appear to primarily arise from patients’ lack of knowledge, understanding, and familiarity with respect to virtual care. Cost, technological access, connectivity, and low digital literacy were challenges for some patients, particularly in rural communities and among older patient population. Patient education and support were critical and needed to be inclusive, easy to understand, and include information regarding privacy, security, consent, and the technology itself. The types of patient education experiences regarded as most helpful included peer support and knowledge sharing among patients themselves.

**Conclusions:**

Beyond the COVID-19 pandemic, virtual care will have a continuing role in enhancing the continuity of care for patients through more convenient access. The education and informational needs of patients are important considerations in promoting the adoption and use of virtual care. Key education and informational needs and strategies were identified to enable and empower patients with the knowledge, digital literacy skills, and support to effectively use virtual care.

## Introduction

The COVID-19 pandemic has had a considerable impact on health care systems worldwide, including the need to continue providing diagnosis, treatment, monitoring, and follow-ups during the pandemic despite major outbreaks and public health restrictions. Virtual care arose as an important solution to enabling ongoing health care delivery services to the public despite the social contact restrictions and limitations to in-person care. Emerging research from the COVID-19 pandemic period suggests that virtual care has been beneficial for health care providers, patients, and the general community [1-3]. Adopting virtual care during COVID-19 has saved on costs associated with personal protective equipment and disinfecting health care spaces [4-6], and enabled patients to stay home who may have otherwise traveled to a hospital and incurred the risk of unnecessary exposure [7,8]. Virtual care adoption also allowed COVID-19 patients remaining at home to continue receiving follow-up and monitoring from providers [6].

Virtual care has been defined as any interaction between patients or clients and members of their circle of care, occurring remotely, using any forms of information and communication technologies with the aim of facilitating or maximizing the quality and effectiveness of patient care [9]. Virtual care has also been referred to as the delivery of health care services digitally or at a distance using information and communication technologies [10]. During the COVID-19 pandemic, various virtual care modalities have been used, with the most common types including synchronous and asynchronous appointments between patients and providers. Synchronous virtual care is communication that occurs live, including telephone and videoconferencing. Asynchronous is communication that does not occur live and may include email, patient portal messages, and e-consults.

Virtual care was used for control and triage during the COVID-19 pandemic, self- and distance monitoring, treatment, and implementation of web-based health services (eg, electronic vaccination records). The use of virtual care during COVID-19 was believed to offer more timely care while minimizing exposure to protect health care providers and patients [4,5,7,10]. By minimizing in-person visits and reducing face-to-face contact among physicians and patients, the use of virtual care may have helped to reduce virus transmission and protect health care providers from infection. Another beneficial aspect of virtual care was that it helped to keep patients informed by enabling access to health information. For instance, during COVID-19, the public had the capability to access data related to COVID-19 transmission [4]. Virtual care also decreased wait times for some patients, particularly for diagnosis, along with facilitating follow-up capabilities for patients who could be monitored from home [4,5].

In general, patients responded positively to virtual care as it enabled more convenient communication with health care providers from their own homes and, for some, reduced travel and increased access [2,3,8]. Imlach et al [11] reported that 91% of patients in their survey study were satisfied with virtual care compared to 92% who expressed satisfaction with in-person care. However, a number of challenges did arise for patients with the move to virtual care. Gaps in digital literacy skills and technological challenges with the use of computers arose for patients unfamiliar with videoconferencing and associated software systems [2,3,12]. The reality is that many patients do experience limited digital literacy and are subsequently unable to use virtual care adequately [7]. Technological challenges for patients are imperative to consider because frustrations can lead to decreased acceptance of virtual care.

Patients have also highlighted data protection as a limiting factor in the adoption of virtual care with concerns about the digital collection of medical information and possible security risks with personal data, especially with the use of web-based, third-party platforms [1-4,10,12]. Some patients may not have the capability to have a quiet, confidential conversation with a health care provider from their home [13,14]. Connectivity challenges also arose as barriers to virtual care use for patients. Internet access is crucial for patients using virtual care, other than telephone calls [1,7,15], and for patients in some rural areas, or of lower socioeconomic status, limited access to good internet connectivity or technology was a real barrier [13,14].

Patient engagement is an important step in the process of developing and implementing health care policy and system change. The Canadian Institutes of Health Research [16] defines patient-oriented research as research that engages patients as partners, focuses on patient-identified priorities, and improves patient outcomes. According to Aubin et al [17], patients have a fundamental right to be engaged in research, and by engaging those who are most affected by health care decisions, patient-oriented research aims to build on patients’ perspectives, needs, priorities and can lead to better transparency and accountability. Patients bring an unique perspective as “experts” from their experiences with the health care system. Engaging patients and examining their experiences with the use of new health care innovations such as virtual care is critical to ensuring that the patient perspective is taken into account in enhancing service delivery. Involving patients in virtual care research is a key way to explore unique solutions because of their experiences as recipients of virtual care [2,3].

Patient education is also another key element in helping patients understand medical information communicated by their health care providers. Patient education is defined as the process of influencing patient behavior and producing the changes in knowledge, attitudes, and skills necessary to maintain or improve health [18]. A goal of patient education is to instill a sense of autonomy in the patient and successful patient education empowers patients to understand, find, consider, and use health services that match their needs and preferences [19]. Effective patient education becomes even more important when new innovations are introduced into health care delivery systems as occurred with the adoption of virtual care appointments during the COVID-19 pandemic. However, a key barrier to achieving a high-level understanding of complex health or technological information can involve patients’ level of health and digital literacy [20]. Understanding the nature and sources of these challenges, and ways to overcome or address, can help in improving patient and health education strategies in the future.

Rogers’ Innovation Diffusion Theory is one of the most popular theories for studying the adoption of IT and has been applied in several studies to conceptualize the adoption of telehealth and virtual care [21-24]. According to this theory, innovation is an idea, process, or a technology that is perceived as new or unfamiliar to individuals. The “attributes of an innovation” can influence the successful diffusion and adoption of a technological innovation like virtual care. Specifically, the “attributes of an innovation” include 5 user-perceived qualities: relative advantage, compatibility, complexity, trialability, and observability. Relative advantage is the degree to which an innovation is perceived as better or improves upon existing practices, while compatibility describes the extent to which an innovation is consistent with the values, past experiences, and needs of the potential adopter. The more an innovation integrates or coexists with these, the greater its prospects for adoption. Complexity involves the degree to which an innovation is perceived to be difficult to understand and use. Innovations with less complexity are more likely to be adopted more quickly, and education that enhances users’ comfort and expertise in using a technological innovation effectively is helpful in facilitating its adoption and effective use. Trialability is the extent to which an innovation can be experimented with on a limited basis, whereas observability describes the degree to which the benefits of an innovation are visible to potential adopters. There have been limited qualitative approaches that have been reported to engage patients in virtual care research and minimal study of the type of educational or informational supports that would be most beneficial to patients in using virtual care. Applying the principles of Rogers’ Innovation Diffusion Theory, the study purpose was (1) to explore patients’ experiences and perspectives with the adoption and use of virtual care during the COVID-19 pandemic and (2) identify the educational and informational needs of patients to inform future strategies for supporting patient use of virtual care.

## Methods

### Recruitment and Data Collection

Phenomenology is a qualitative research methodology, which emphasizes participants’ perceptions, feelings, and experiences with the goal to search for the “essential structures” of the phenomenon by interviewing, in depth, individuals who have experienced the phenomenon [25]. A focus group interview was conducted in February 2022, with a purposive sample of patient representatives to explore patient experiences and perceptions of the use of virtual care during the COVID-19 pandemic. The use of the phenomenological focus group interview method has been supported by a number of authors for use in health research [26,27]. Descriptive phenomenology was adopted to allow for a focus on a description of respondents’ experiences in using virtual care during the COVID-19 pandemic and the commonalities, which existed across the participants’ perspectives [28].

Potential focus group interview respondents were identified with the assistance of the patient engagement lead with Newfoundland and Labrador (NL) Support using their Patient Advisory Council. NL Support is the NL Strategy for Patient-Oriented Research Support unit housed within the Faculty of Medicine at Memorial University. The Patient Advisory Council is comprised of 25 patients, representing a cross-section of the population of the province of NL. The patient engagement lead circulated the focus group information and description of the study purpose to the patient advisory council. If a patient representative was interested in participating in the focus group, they were invited to contact the patient engagement lead and consent to have their contact information shared with the study investigators. The only criterion for inclusion in this research study was that they had personally experienced virtual care at least once during the pandemic.

Fifteen patient representatives expressed interest in the focus group and agreed to be contacted. Thirteen patient representatives responded to a follow-up invitation by the study investigators. The period for this study was very narrow, and a poll was conducted with these 13 patient representatives to determine the best date and time to conduct the focus group. Seven patient representatives subsequently agreed to participate, and on the day of the focus group, 2 participants notified investigators they were no longer able to participate. Consequently, 5 patient representatives participated in the focus group interview.

The focus group was conducted for 60 minutes via videoconference and was recorded with the respondents’ consent. Multimedia Appendix 1 outlines the focus group interview questions. As discussed, Rogers’ Innovation Diffusion Theory was applied as a theoretical framework, and more specifically, we used the concepts underlying the attributes of the innovation—“complexity,” “relative advantage,” and “compatibility”—in constructing the focus group questions. Respondents were provided with the focus group question script prior to the focus group.

### Data Analysis

Qualitative data were analyzed using the thematic analysis technique—a common form of analysis in qualitative research that emphasizes pinpointing, analyzing, and recording patterns (or “themes”) within data [29].

### Ethics Approval

This study was submitted for human subject research ethics review. The Newfoundland and Labrador Health Research Ethics Board reviewed and provided approval for this study (2021.239). Participants were provided with a study consent form via email, asked to review the form, and invited to contact study investigators if they wished with any questions prior to signing and returning the form.

### Informed Consent

All participants provided informed written consent prior to participating in the study and were asked to verbally reaffirm their consent at the start of the focus group.

## Results

### Overview

The focus group respondents included 3 men and 2 women, with an age range of 20-65 years. Respondents represented 3 of the 4 health care regions in the province: Eastern Health (n=3), Western Health (n=1), and Labrador-Grenfell Health (n=1). Each of the patient representatives had at least 1 personal experience with virtual care during the pandemic.

Respondents in the focus group discussed their experiences and perspectives about the use of virtual care in accessing health care services and interacting with health care providers. Rogers’ attribute of “relative advantage” is described as the degree to which an innovation is perceived as better or improves upon existing practices, while “compatibility” describes the extent to which an innovation is consistent with the values, past experiences, and needs of the potential adopter. Respondents acknowledged that COVID-19 has demonstrated the importance of virtual care for accessing health care services, and while in-person appointments are ideal, virtual care is a beneficial addition to the health care system. Positive language was used during the discussion to describe virtual care, including “*excellent*” and *“wonderful*,” and respondents highlighted the usefulness of virtual care not only with increasing accessibility but also for mitigating barriers to accessing services. Some of these barriers included the distance, time, and cost of travel to receive health care from more rural and remote areas of the province. Reducing expenses with respect to time, money, travel, and stress was a key part of the discussion. The option of virtual care enables patients to access health care services while balancing their schedules, including work commitments and childcare availability.

It is really convenient in expanding into other options. It is really great to fit into peoples’ busy schedules. Working from home, a lot of peoples’ child care responsibilities have changed so they may not be able to leave their houses. So they may be able to have an appointment during a lunch break while working from home.

P5

Better personal healthcare because it gives some patients access to healthcare who may not seek it for whatever reason; travel cost, fear, and accessibility. Patients who would normally not have access to specialists, this will give them an option.

P4

A lot of people have to travel to see a specialist. It is very expensive, if virtual care can help with that and reduce the expense, it is that much better.

P3

Respondents also discussed the importance of “*options*” within the health care system. Virtual care should not replace in-person care but instead complement in-person services. It has a place in the system but should not be the only option. For instance, respondents described how during the pandemic some physicians offered a mix of services. This could mean holding virtual appointments in the morning, and in-person services in the afternoon. Additionally, some physicians may have requested patients come in to see them following a phone conversation. Another key consideration was the mode of virtual care offered. Although phone appointments were normalized during the pandemic, respondents felt they were not good enough for everyone. Video appointments enabled physicians to observe patients’ body language, which respondents highlighted as an important aspect of diagnosis.

For example, my physician offered a schedule. Mornings were for calls and the afternoons were for in-person appointments. So that was nice because you could schedule yourself in where you thought it was best for your situation. It provided that option which was really good. It should be used to supplement, it shouldn’t replace anything, but I think having the options is fantastic and should continue.

P5

Phone calls are not good enough for everyone, although they might work for some. We need video options, not just phone calls, because of the importance of body language for diagnosis.

P3

### Barriers and Challenges

#### Overview

Rogers’ attribute of “complexity” is described as the degree to which an innovation is perceived to be difficult to understand and use, with greater complexity being a larger barrier or challenge to adoption. Despite the benefits of virtual care, there were a number of key barriers and challenges for patients in adopting and using virtual care, including “fear of the unknown,” “lack of communication,” “technology and connectivity challenges,” “cost,” and “comfort.”

#### Fear of the Unknown

The potential “fear of the unknown” for patients was acknowledged, resulting from a lack of understanding of technology. Patients may experience an associated concern for their confidentiality and privacy or question whether virtual care is as effective as in-person services, considering the absence of a physical examination. This is especially relevant for phone calls, where providers are unable to see patients in any capacity.

You are unsure if it is going to be as effective. If you were having a physiotherapy appointment and you are trying to show your arm and how it is moving. They might not think it is going to work if we can’t actually have that hands on care. The concerns of effectiveness is a big barrier.

P5

#### Lack of Communication

Lack of communication regarding the benefits of virtual care was highlighted as a barrier to patients’ understanding. There was a lack of adequate communication with patients about the effectiveness of virtual care in comparison to in-person care. Respondents believed that greater communication and information about the benefits of virtual care would have been useful. Patient consent was also raised as a priority and ensuring patients have consent information, and can consent, as required.

#### Technology and Connectivity Challenges

Respondents also highlighted connectivity as a key barrier. Patients may experience technical difficulties, or even “*dropped sessions*,” due to connectivity issues and may not know what to do. Technology support was highlighted as an important consideration to assist patients. Also discussed was that different software may be used across different regional health authorities and patients may have different devices available to them. Technology compatibility could be a barrier for patients, as some software works better with certain devices. Respondents suggested the need to test software in regions experiencing connectivity issues to determine which software works best.

For both patients and providers the use of technology and technology supports is a big barrier. There have been so many dropped sessions in rural areas.

P2

We use a variety of different platforms for virtual care, Jabber, EMR, Webex. When you are trying to provide these home based appointment, you are using different or a variety of mobile devices. Each platform works better with a different device. Knowing that information is critical to having a successful virtual care visit. Sometimes it is better to have something in place to test with a client before they even have their appointment. To make sure everything works. Testing this and letting your clients know what works best in each case. We are not there, but we need to get there.

P1

#### Cost

The required technology for virtual care can introduce an expense for patients, which acts as a barrier to accessing health care. Respondents highlighted the expense of technology required to access virtual care services, such as iPads and mobile plans. Often those who need care the most may be in an underprivileged position, for instance, experiencing low income.

#### Comfort

Respondents discussed how a patient’s comfort level could also be a barrier toward using virtual care. Lack of exposure to virtual care may reduce patients’ comfort with accessing these services. Videoconferencing appointments essentially bring health care providers into the homes of patients and respondents highlighted concerns over “*tidying their homes*” or the presence of “*dirty dishes*.”

### Patient Education and Informational Resources and Strategies

#### Overview

Respondents were also asked to describe informational and educational resources they believed to be most helpful to patients. Ongoing education and support for patients were deemed critical with a key requirement that patients understand what virtual care means and have a good appreciation for what a videoconferencing appointment would be like from start to finish. Patients should also be aware of anything they need to do to prepare for a virtual care appointment.

#### Understanding the Technology

An aspect of preparation is having knowledge of what platforms and technology options are available. Patients need to know what technology will be required to access virtual care and how to connect to the appointment. For instance, downloading a videoconferencing platform or a mobile application may be necessary for a video appointment. Preparation can reduce anxiety and ensure patient comfort during the appointment. Information on the privacy and security of software would be helpful for patients as well. Respondents felt that the patients should know how their information would be protected.

Need to provide information to patients on the different modes that are offered. That there is an option for a phone call, in-person, and videoconferencing and just what those platforms would look like. A ‘how to’ for the technology piece of it.

P5

We are facing a population that is not computer literate, seniors. It is so important to provide education and support. This will hopefully reduce anxiety and ensure the patient is more comfortable during the appointment.

P3

Information about the different tools and ways that virtual care can be provided. Using local libraries to help educate and provide support may help make the use of the technology more comfortable for people.

P4

Information about their privacy and how it is protected, How is my privacy protected? Don’t just tell me it is, tell me how it is protected.

P2

#### Education and Communication Methods

Respondents discussed the importance of an education and communication plan in promoting the adoption and use of virtual care with patient populations. Web-based training and information can be beneficial for patients, and it was felt that much information and resources may already be available through the internet but could be collated with patients being directed to this information. However, it was acknowledged that the patient population is a broad demographic (eg, age, ethnicity, and comfort with technology), so it is important to be aware of such differences and have multiple information outlets to address educational needs. Existing institutions, such as libraries, could be used to provide information about virtual care and how to access and set up videoconferencing appointments. They could help provide information about privacy concerns and assist with patient comfort if they are anxious about holding appointments in their homes. Collaboration with patient and community groups, such as Indigenous communities, can assist in providing information to their communities.

#### Peer Support and Community Knowledge Sharing

Respondents highlighted the benefit of peer support. People with lived experience are resources for educating patients and could use their experience to share knowledge with other patients. Success stories are useful in encouraging patient comfort with using virtual care. Community-based education targeting the general population can also be facilitated through open houses to the public. Respondents also discussed the importance of community-based organizations, such as senior groups, in providing members with opportunities for educational experiences.

Using patients to support development and imparting education, patients learning from other patients’ experiences makes a big, big difference. If you have had someone with a chronic disease and they have gone through virtual care, what a great resource to use for the next person you are offering virtual care to.

P2

I think testimonials are a very important part of moving forward. If you have people who have had success [using virtual care] and if they want to tell others about their success stories, I think that would be wonderful.

P1

## Discussion

### Principal Findings

Applying principles of Rogers’ Innovation Diffusion Theory, the study purpose was (1) to explore patients’ experiences and perspectives with the adoption and use of virtual care during the COVID-19 pandemic and (2) to identify the educational and informational needs of patients to inform future strategies for supporting patient use of virtual care. Specifically, focus group interview questions were intended to explore the advantages and complexities of using virtual care in receiving health care services, as well as the compatibility of virtual care appointments in meeting patient needs. We were also interested in identifying specific education or informational needs from the patient perspective that could be addressed in supporting patient use of virtual care technologies in the future.

In general, respondents reported positive perceptions of the use of virtual appointments during the COVID-19 pandemic and felt that virtual care was a very useful adjunct to health care delivery services. For some, virtual care provided more convenient access to appointments with their health care provider. These findings reflect the findings from other studies of virtual care during the pandemic [2,3]. Patients respond positively to virtual care with travel reduced and accessibility increased, allowing some individuals the convenience of communicating with health care providers from their own homes [8]. Considering clinical outcomes, small-scale studies comparing the outcomes of in-person and virtual mental health care indicate that virtual care could be as effective as in-person care [13]. The shared decision-making process, which is beneficial in patient-centered care, was also preserved with virtual care appointments during the COVID-19 pandemic [30]. Patient-centered virtual care allowed providers to treat patients based on their personal preferences, and this was especially true for synchronous appointments [31]. Furthermore, patient-centered care enabled by virtual care technology is empowering. Patients are more involved in their own care plan, increasing their understanding and improving their health in a collaborative way. The result is increased empowerment associated with independence and self-management of their health [12].

Respondents did identify several key barriers and challenges that patients encountered in adopting and using virtual care. Some of these barriers and challenges arise from patients’ lack of knowledge about virtual care, which can contribute to a fear of the unknown experienced by patients. Patients unfamiliar with virtual care software could be apprehensive about it, leading to resistance to virtual care adoption [1,4,14]. Patients with no previous experience with virtual care could be more likely to believe their specific needs are not appropriate for virtual care appointments [15]. Respondents indicated that there is a need for patients to know what virtual care means, including what a virtual care appointment involves and who it benefits. Patient education can assist in increasing the understanding of virtual care use and other associated health benefits [7,9,15,30]. Respondents also described specific patient education experiences that might be most helpful for patients in learning about virtual care. Respondents highlighted the benefit of peer support and knowledge sharing among patients themselves. It was acknowledged that people with lived experience could be resources for educating patients and community-based knowledge sharing was also encouraged.

Due to the nature of virtual care, connectivity was also an issue of importance. Any connectivity challenges can serve as barriers to virtual care use for patients. Internet access is crucial for patients using virtual care other than telephone calls [1-3,7,15]. Patients in some rural areas, or of lower socioeconomic status, may not have the capability to access good internet connectivity or technology to access virtual care [13,14]. Patient concern around data security and privacy protection was another area that emerged from the focus group discussion. Studies suggest that patients highlight data protection as a limiting factor in the adoption of virtual care. Virtual care use implies the necessity of the digital collection of medical information. The concern is that this collection could lead to a security risk for personal data, especially with the use of web-based, third-party platforms [1,4,10,13].

Patients have also expressed concerns regarding the nature of virtual care and specifically, the lack of physical examinations [2,3,8]. There could be reluctance, or lack of privacy, to use videoconferencing to show certain parts of the body [14]. Digital health literacy is closely related to health literacy and refers to a patient’s ability to use health technology to interact with their own health care provider and the health care system at large. Overall, comfort and confidence in using technology can be impacted by a lack of understanding and familiarity with virtual care. Respondents felt that ongoing patient education and support were important regarding privacy, security, and the types of technology necessary for virtual care. It is also important to ensure patients have information on virtual care consent. Additionally, patients may experience challenges facilitating communication and establishing a good provider–patient relationship [1,8,13]. Respondents highlighted the importance of special attention to the needs of some groups. Attentiveness and inclusion of the specific needs of groups, such as seniors and Indigenous communities, are important to ensure they receive the information most relevant to them.

This focus group study represented the views of patient representatives with specific cultural backgrounds, characteristics, and ages from a particular geographic region in Canada. Similarly, the characteristics of the health care system were unique to this region and may not be reflective of other health care systems on a wider level. The number of focus group participants was also limited; therefore, the viewpoints and perspectives reflected in the study findings should be interpreted in light of these limitations. Nonetheless, the key themes emerging from the focus group interview do reflect and confirm findings that have emerged from other studies on the topic. There are several areas of future inquiry that would add to our growing understanding of the patient experience with virtual care. Further examination of the relationship between patient attitudes and confidence toward virtual care and patient satisfaction with virtual care experiences would be useful. In addition, more exploration of the influence of gender and diversity-based characteristics on patient adoption and use of virtual care would be helpful, as well as the barriers and enablers for older patient populations in using virtual care. Together, further study of this field would increase our understanding of factors influencing virtual care adoption and use from the patient’s perspective.

### Conclusions

In this qualitative study, we sought to explore patients’ experiences with the use of virtual care during COVID-19 and identify the educational and informational needs of patients to better inform patient educational strategies surrounding virtual care in the future. Beyond the COVID-19 pandemic, patients believe that virtual care will have a continuing role in enhancing continuity of care through more convenient access. A number of barriers and challenges to patient use of virtual care were identified. Patients also emphasized that the educational and informational needs of patients are important considerations in promoting the adoption and use of virtual care. By adapting patient educational strategies to patients’ needs, future education and informational supports and resources are more likely to be successful in enabling patient use of virtual care.

## Appendix

Multimedia Appendix 1 Patient Focus Group Script.

## Abbreviations

NL: Newfoundland and Labrador

## Data Availability

The data generated and analyzed during the current study are available from the corresponding author on reasonable request.
