# Supplementary material for: Patient Experiences With Virtual Care During the COVID-19 Pandemic: Phenomenological Focus Group Study
Source: JMIR Form Res. 2023 May 1;7:e42966. doi: 10.2196/42966 (PMC10154021; doi:10.2196/42966)
Supplement: Multimedia Appendix 1 [file formative_v7i1e42966_app1.docx]

**Appendix A**

**Patient Focus Group Script**

1. What are your general thoughts about the use of “Virtual Care” in accessing health care services and interacting with physicians and healthcare professionals for your health care?
2. What are key barriers and challenges for patients in adopting and using “Virtual Care” to access health care services and interact with physicians and healthcare professionals for their health care?
3. What informational and/or educational resources would be most helpful to patients in understanding and becoming more comfortable with using “Virtual Care” for receiving health care services?
4. What do you feel patients need to ‘know’ or ‘do’ to use “Virtual Care” in accessing health care services and interacting with physicians and healthcare professionals?
5. Are there particular “patient education” experiences that might be most helpful for patients in learning about “Virtual Care”?
